# Ferroptosis-related gene signature correlates with the tumor immune features and predicts the prognosis of glioma patients

**DOI:** 10.1042/BSR20211640

**Published:** 2021-12-07

**Authors:** Yan Hu, Zewei Tu, Kunjian Lei, Kai Huang, Xingen Zhu

**Affiliations:** 1 Department of Neurosurgery, The Second Affiliated Hospital of Nanchang University, Nanchang, Jiangxi 330006, P.R. China; 2 Institute of Neuroscience, Nanchang University, Nanchang, Jiangxi 330006, P.R. China; 3 Jiangxi Key Laboratory of Neurological Tumors and Cerebrovascular Diseases, Nanchang, Jiangxi 330006, P.R. China; 4 East China Institute of Digital Medical Engineering, Shangrao, Jiangxi 334000, P.R. China

**Keywords:** Clinical nomogram, Ferroptosis, Glioma, Prognostic signature, Tumor immunity

## Abstract

**Background**: Glioma is a malignant intracranial tumor and the most fatal cancer. The role of ferroptosis in the clinical progression of gliomas is unclear.

**Method**: Univariate and least absolute shrinkage and selection operator (Lasso) Cox regression methods were used to develop a ferroptosis-related signature (FRSig) using a cohort of glioma patients from the Chinese Glioma Genome Atlas (CGGA), and was validated using an independent cohort of glioma patients from The Cancer Genome Atlas (TCGA). A single-sample gene set enrichment analysis (ssGSEA) was used to calculate levels of the immune infiltration. Multivariate Cox regression was used to determine the independent prognostic role of clinicopathological factors and to establish a nomogram model for clinical application.

**Results**: We analyzed the correlations between the clinicopathological features and ferroptosis-related gene (FRG) expression and established an FRSig to calculate the risk score for individual glioma patients. Patients were stratified into two subgroups with distinct clinical outcomes. Immune cell infiltration in the glioma microenvironment and immune-related indexes were identified that significantly correlated with the FRSig, the tumor mutation burden (TMB), copy number alteration (CNA), and immune checkpoint expression was also significantly positively correlated with the FRSig score. Ultimately, an FRSig-based nomogram model was constructed using the independent prognostic factors age, World Health Organization (WHO) grade, and FRSig score.

**Conclusion**: We established the FRSig to assess the prognosis of glioma patients. The FRSig also represented the glioma microenvironment status. Our FRSig will contribute to improve patient management and individualized therapy by offering a molecular biomarker signature for precise treatment.

## Introduction

Malignant glioma is the most invasive and lethal tumor of the human central nervous system [1]. Based on the World Health Organization (WHO) classification method, gliomas are classified into grades I–IV. Grades II–IV have received extensive attention by researchers because of their lack of response to clinical treatment [2,3]. Currently, the mainstream therapy of gliomas includes surgical resection combined with chemotherapy and/or radiotherapy but the therapeutic effects and survival of glioma patients are still unsatisfactory. The lack of understanding of the intratumor microenvironment and molecular characterizations of oncocytes may be partly responsible. Thus, uncovering the molecular pathogenesis of gliomas and transferring targeted mechanisms to clinical applications are essential and meaningful for developing novel treatment methods for patients with gliomas.

The pathways regulating cell death include the programmed death pathway, autophagy, apoptosis, and pyroptosis [4]. Dixon et al. [5] first proposed ferroptosis as a novel cell death in 2012. Unlike autophagy and apoptosis, ferroptosis is an iron-dependent and reactive oxygen species (ROS)-reliant cell death pathway and is characterized by a series of cytological changes, including cell volume contraction, increased lipid peroxidation and increased mitochondrial membrane density (mitochondrial shrinkage). As reported previously, ferroptosis plays a vital role in multiple human diseases like stroke [6], brain trauma [7] and renal failure [8], and its role in tumorigenesis and cancer development is currently being studied comprehensively.

Numerous regulators have been reported to be involved in the process of ferroptosis, and play pivotal roles in tumor progression in tumors such as hepatocellular carcinoma, colorectal cancer, gastric cancer, and ovarian cancer [9–12]. However, the literature lacks a comprehensive analysis of ferroptosis regulators in glioma in correlation with different clinicopathological characteristics, their underlying functions in glioma malignant progression, and their potential prognostic predictive value.

In the present study, we first assessed the associations between the aberrant expression of 28 ferroptosis-related genes (FRGs) and the clinical and molecular characteristics of glioma patients in the Chinese Glioma Genome Atlas (CGGA) (*n*=306) and The Cancer Genome Atlas (TCGA) (*n*=628) datasets. Next, we screened for prognostic FRGs using univariate Cox regression analysis and constructed a reliable and robust prognostic FRG-signature using least absolute shrinkage and selection operator (Lasso) analysis. Furthermore, the FRG-signature was validated as an independent prognostic factor for glioma patients and a nomogram model was built based on the FRG-signature, WHO grade, and patient age for clinical application. In addition, tumor immune characterizations were found to be highly associated with our FRG-signature in gliomas. Our study provides novel insight into the functions of FRGs in glioma immune infiltrations and provides a rationale for future clinical applications.

## Methods

### Data acquisition and processing

Two public repositories, the CGGA (http://www.cgga.org.cn/) and the Genomic Data Commons Data Portal (GDC; https://portal.gdc.cancer.gov/), were searched for mRNA expression and clinical data of glioma patients to be included in the present study. In total, 934 glioma patients treated by surgical excision, living longer than 30 days from diagnosis and accessible for WHO grade information were included. Relevant transcriptome sequencing data, and clinical and molecular data were downloaded for further bioinformatics and statistical analyses. For the two RNA-seq cohorts, original data in the Fragments Per Kilobase of transcript per Million (FPKM) format were transformed to the Transcripts Per Kilobase Million (TPM) format using an algorithm described in a previous study [13,14]. The baseline clinicopathological information of glioma patients included in the present study was listed in the Table 1.Twenty-eight FRGs, including ROS proto-oncogene 1 (*ROS1*, receptor tyrosine kinase), acyl-CoA synthetase long chain family member 4 (*ACSL4*), apoptosis inducing factor mitochondria associated 2 (*AIFM2*), activating transcription factor 4 (*ATF4*), BRCA1-associated protein 1 (*BAP1*), BCL2 apoptosis regulator (*BCL2*), beclin 1 (*BECN1*), cyclin-dependent kinase inhibitor 1A (*CDKN1A*), cysteine dioxygenase type 1 (*CDO1*), cystathionine γ-lyase (*CTH*), ferritin heavy chain 1 (*FTH1*), ferritin light chain (*FTL*), glutamic-oxaloacetic transaminase 1 (*GOT1*), glutathione peroxidase 4 (*GPX4*), heat shock protein family B member 1 (*HSPB1*), vir-like m6A methyltransferase associated (*KIAA1429*, also known as VIRMA), nuclear receptor co-activator 4 (*NCOA4*), nuclear factor, erythroid 2 like 2 (*NFE2L2*), NFS1 cysteine desulfurase (*NFS1*), OTU deubiquitinase, ubiquitin aldehyde binding 1 (*OTUB1*), solute carrier family 11 member 2 (*SLC11A2*), solute carrier family 1 member 5 (*SLC1A5*), solute carrier family 40 member 1 (*SLC40A1*), solute carrier family 7 member 11 (*SLC7A11*), transferrin (*TF*), transferrin receptor (*TFRC*), tumor protein p53 (*TP53*), and zinc finger E-box binding homeobox 1 (*ZEB1*) were defined from a preceding publication [15].

**Table 1:** Clinicopathological information of glioma patients included in the present study

Clinicopathological factor	CGGA cohort (*n*=306)	TCGA cohort (*n*=628)
Age (years)		
≤40	134 (43.79%)	241 (38.38%)
>40	172 (56.21%)	387 (61.62%)
Gender		
Male	189 (61.76%)	362 (57.64%)
Female	117 (38.24%)	266 (42.36%)
IDH mutation		
Yes	167 (54.58%)	384 (61.15%)
No	139 (45.42%)	235 (37.42%)
NA	0 (0.00%)	9 (1.43%)
1p/19q codeletion		
Yes	63 (20.59%)	152 (24.20%)
No	239 (78.10%)	470 (74.84%)
NA	4 (1.31%)	6 (0.96%)
WHO grade		
II	97 (31.70%)	219 (34.87%)
III	74 (24.18%)	243 (38.69%)
IV	135 (44.12%)	166 (26.43%)

Abbreviations: IDH, isocitrate dehydrogenase; 1p/19q, the short arm of chromosome 1 and the long arm of chromosome 19.

### Sample collection

Six pairs of glioma core samples and para-cancerous tissues were collected to verify the protein expression levels of HSPB1 and SLC1A5. Samples were resected from six glioma patients (two WHO grade II, one WHO grade III, and three glioblastomas (GBMs)) in the Neurosurgery Department of The Second Affiliated Hospital of Nanchang University from 2020 to 2021. These glioma core samples and para-cancerous tissues were frozen in liquid nitrogen. This research has been approved by the Medical Ethics Committee of The Second Affiliated Hospital of Nanchang University. The processes of sample collection and employ were fully in accordance with the approved guidelines. Informed consents were gained from each glioma patient.

### Western blot and antibody

RIPA lysis buffer with 1% PMSF was used to extract total sample protein, and protein concentrations were determined by BCA assay (Beyotime, China). A total of 15 μg total protein of each sample was added in SDS/PAGE gel and separated by electrophoresis and the separated proteins were transferred to PVDF membranes (Millipore, MA, U.S.A.). Then the PVDF membranes were blocked in 10% skim milk at 25°C for 1 h, then cropped and incubated with anti-SLC1A5 (1:1000, 20350-1-AP, Proteintech, China), anti-HSPB1 (1:1000, 18284-1-AP, Proteintech), anti-GAPDH rabbit polyclonal antibody (1:4000, 10494-1-AP, Proteintech) at 4°C overnight, respectively. The HRP-conjugated Affinipure Goat Anti-Rabbit IgG (1:4000, SA00001-2, Proteintech) were used to incubate the PVDF membranes 2 h at room temperature.

The stripes of interest proteins were developed with enhanced chemiluminescence (ECL, Thermo Fisher Scientific, 32106, U.S.A.) reagents by means of GV6000M (GelView 6000pro, China). The gray intensity of HSPB1 and SLC1A5 were calculated using the ImageJ software (National Institutes of Health, U.S.A.) and standardized to the intensity of corresponding GAPDH.

### Construction of the ferroptosis-related signature

The CGGA cohort acted as the training cohort for establishing the ferroptosis-related signature (FRSig). First, we used univariate Cox regression to evaluate the prognostic roles of the 28 FRGs. Then, the expression of 22 FRGs with statistical significance (|hazard ratio (HR)| > 1.00 and *P*<0.05) were subjected to Lasso Cox regression, which is a classical method used to construct a high-dimension expression matrix, and a prognostic predictive FRG-signature was developed based on the CGGA cohort. Finally, a prognostic model was established based on ten FRGs; the formula was described as:

$$Risk\;score\;=\;\sum_{i=1}^{n}\;Coef_{i}\cdot x_{i}$$

in which *Coef_i_* indicates the coefficients, *x_i_* is the TPM value of each FRG.

### Biological process enrichment and pathway analysis

Before biological process enrichment and pathway analysis, differentially expressed genes (DEGs) between the low- and high-risk glioma subgroups were identified using the ‘limma’ package [16] under the standard of |log2(fold change) | > 1 and a *P*-value <0.05. A total of 1632 DEGs were identified and were included in the functional enrichment analysis using the webtool ‘Metascape’ [17], a gene annotation and analysis resource, which includes Canonical Pathways, Reactome Gene Sets, Gene Ontology (GO) Biological Processes, and the Kyoto Encyclopedia of Genes and Genomes Pathway (KEGG pathway). Gene set enrichment analysis (GSEA) [18], a computational method that determines whether an *a priori* defined set of genes shows statistically significant, concordant differences between two biological states, was used to evaluate the significantly enriched hallmarks in the high-risk glioma patients.

### Assessment of cell infiltration of the tumor microenvironment

We performed single-sample gene set enrichment analysis (ssGSEA) [18], an extension of GSEA, to calculate an enrichment score for each sample using a specific gene set, which quantified the level of cell infiltration or immune-related activities in the glioma TME. Immune-related gene sets used in our analysis were downloaded from the MSigDB website (https://www.gsea-msigdb.org/). Using these gene sets, relative enrichment scores (ESs) were calculated based on the levels of corresponding abundance of non-neoplastic cell infiltrating or immune-related activities in each glioma sample.

### Establishment of an FRSig-based nomogram model

The prognostic nomogram model was established and validated by multivariate Cox regression method using the ‘rms’ R package. Considering the prognostic power and clinical accessibility of factors with statistical significance in the multivariate Cox regression analysis, age, WHO grade, and the FRSig risk score were incorporated in the nomogram. Calibration analysis of the prognosis predictive value of the nomogram were carried out using the ‘calibrate’ function of the ‘rms’ package. To evaluate the clinical translational potential of the nomogram, we used the decision curve analysis (DCA) to calculate the clinical benefits of different factors.

### Statistical analysis

The Student’s *t* test and one-way ANOVA test were used to compare differences of two or three glioma subgroups, respectively, and paired *t* test was used to compare the protein expression difference of HSPB1 and SLC1A5 between paired glioma samples and para-cancerous tissues. In consideration of the prognostic role of the FRSig, survival curves of the prognostic analysis were determined using the Kaplan–Meier method and statistical significance was established using the two-sided log-rank test. We chose the most statistically significant cut-off point using the ‘surv-cutpoint’ function of R package ‘survminer’. We applied the univariate Cox regression model to identify the prognostic role of each FRG and calculate the HR of FRGs associated with survival. To estimate the prognostic predictive performance of the FRSig, time-dependent receiver operating characteristic curve (ROC) model analysis was carried out to calculate the area under the curve (AUC) using the ‘timeROC’ package. A two-sided statistical *P-*value <0.05 was considered as statistically significant in the present study.

## Results

### Correlations between expression levels and clinicopathological features in gliomas

**Figure 1:**
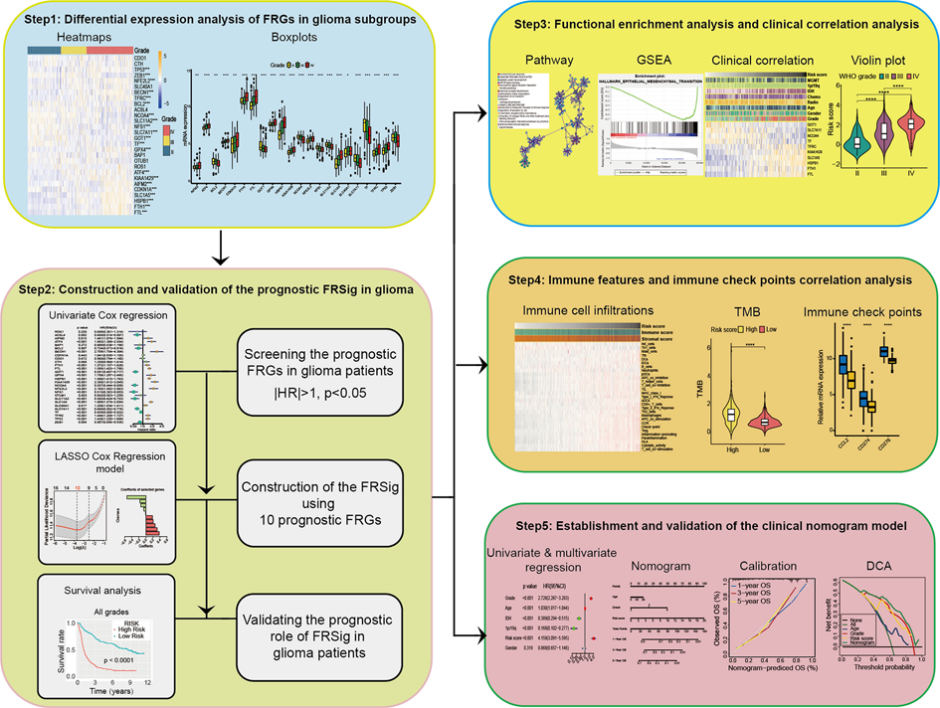
The workflow chart of our study

**Figure 2:**
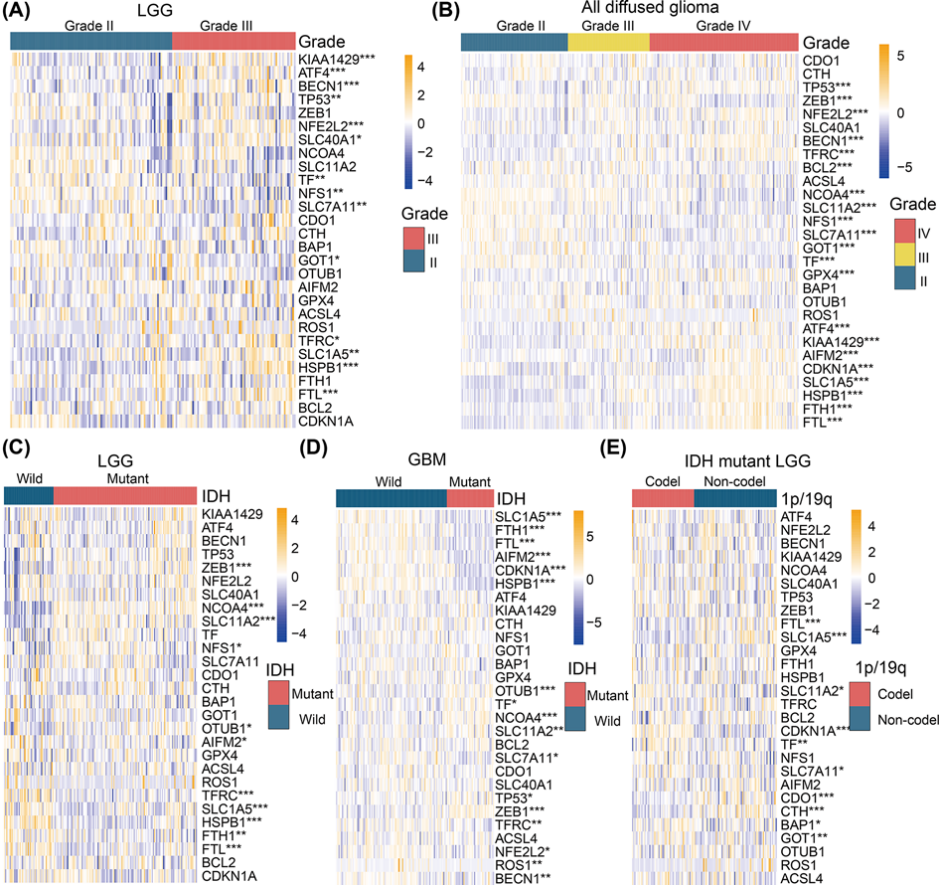
Heatmaps present different expression levels of FRGs among distinct glioma subgroups (**A**) Differential expression levels of 28 FRGs between WHO grade II LGGs and grade III LGGs. (**B**) Differential expression levels of 28 FRGs among WHO grade II, III, and IV gliomas. (**C**) Differential expression levels of 28 FRGs between IDH-wildtype and IDH-mutant LGGs. (**D**) Differential expression levels of 28 FRGs between IDH-wildtype and IDH-mutant GBMs. (**E**) Differential expression levels of 28 FRGs between 1p/19q co-deletion and 1p/19q non-codeletion IDH-mutant LGGs. *P<0.05, **P<0.01, ***P<0.001.

The whole design of our study was described as Figure 1. Considering the pivotal biological functions of each ferroptosis regulator in tumorigenesis and development, we investigated the relationships between each individual ferroptosis regulator and the pathological features of gliomas, including WHO grade, isocitrate dehydrogenase (IDH) mutation status, and 1p/19q co-deletion status. The expression of each ferroptosis regulator and associated WHO grades are presented as heatmaps (Figure 2A,B). The expressions of most ferroptosis regulators are significantly associated with WHO grades. Next, we evaluated the relationship between the expression level of each individual ferroptosis regulator and IDH mutation status in lower grade gliomas (LGG, Figure 2C) and GBMs (Figure 2D), respectively. The results showed that expression of *ZEB1*, *NCOA4*, *SLC11A2*, *TFRC*, *SLC1A5*, *HSPB1*, *FTH1*, and *FTL* differed significantly between LGGs with mutated IDH and wildtype-IDH LGGs in the CGGA datasets (Figure 2C). *SLC1A5*, *FTH1*, *FTL*, *HSPB1*, *NCOA4*, *SLC11A2*, *ZEB1*, and *TFRC* genes were also differentially expressed in GBM patients with and without IDH mutation in the CGGA dataset (Figure 2D). In patients with LGG harboring IDH mutation, we observed that the expression of *FTL*, *SLC1A5*, *CDKN1A*, *CDO1*, *CTH*, and *GOT1* was highly correlated with the status of 1p/19q co-deletion (Figure 2E).

**Figure 3:**
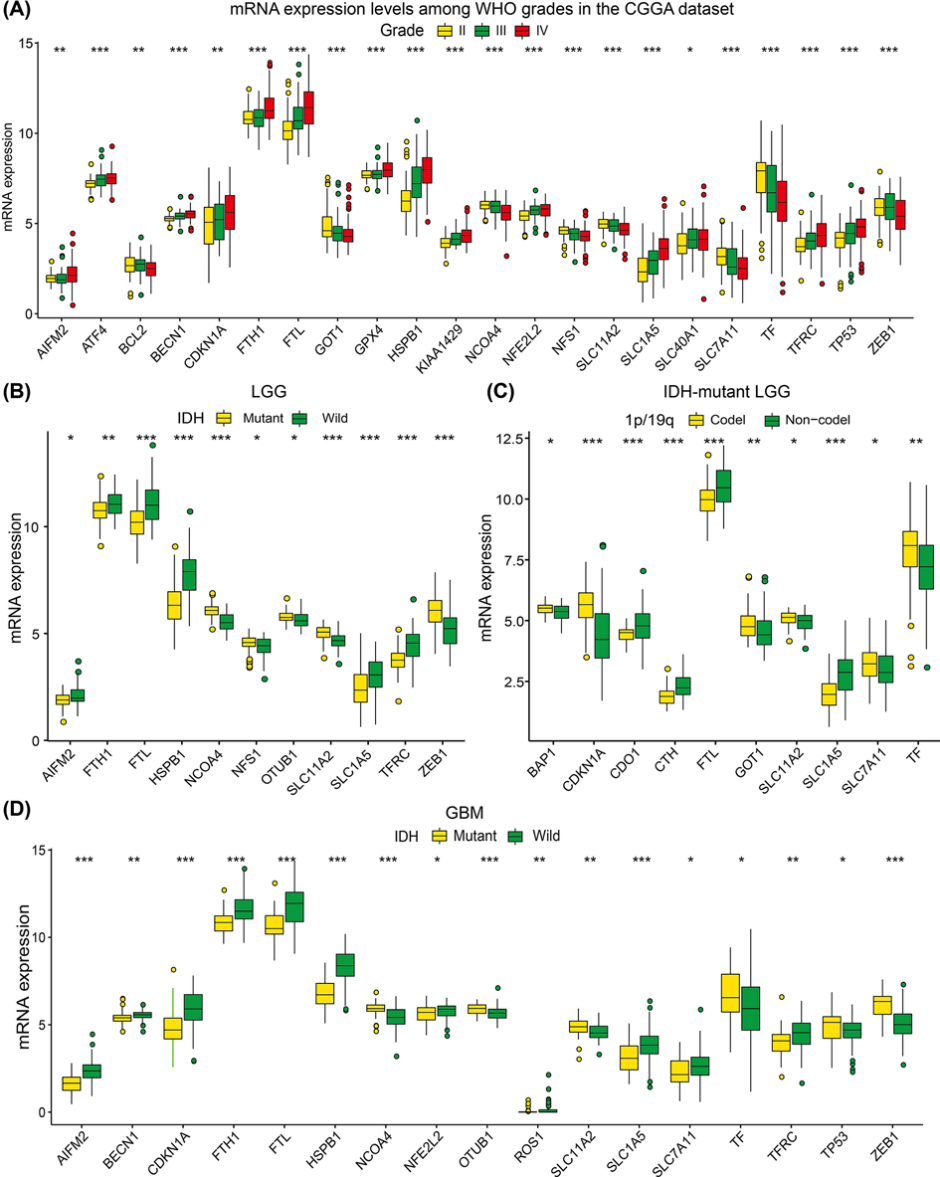
Boxplots showed the statistical significance of differentially expressed FRGs among different clinicopathological features of gliomas (**A**) Twenty-two FRGs were differentially expressed among different grade gliomas significantly. (**B**) Eleven FRGs were significantly differentially expressed between IDH-wildtype and IDH-mutant LGGs. (**C**) Ten FRGs were significantly differentially expressed between 1p/19q co-deletion and non-codeletion IDH-mutant LGGs. (**D**) Seventeen FRGs were significantly differentially expressed between IDH-wildtype and IDH-mutant GBMs. *P<0.05, **P<0.01, ***P<0.001.

Significant correlations between WHO grades and expression of *BECN1*, *TP53*, *NFE2L2*, *SLC1A5*, *HSPB1, FTL*, *GOT1*, *NFS1*, *SLC11A2*, *SLC7A11*, and *TF* were also confirmed by quantitative analyses using the CGGA datasets (Figure 3A). For LGGs, AIFM2, FTH1, FTL, HSPB1, SLC1A5 and TFRC were upregulated in IDH-wild LGGs and NCOA4, NFS1, OTUB1, SLC11A2 and ZEB1 were downregulated (Figure 3B). And in IDH-mutation LGGs, CDO1, CTH, FTL and SLC1A5 were overexpressed in 1p/19q non-codeletion samples and BAP1, CDKN1A, GOT1, SLC11A2, SLC7A11 and TF were downregulated in 1p/19q non-codeletion samples (Figure 3C). Additionally, AIFM2, BECN1, CDKN1A, FTH1, FTL, HSPB1, NFE2L2, ROS1, SLC1A5, SLC7A11 and TFRC were upregulated in IDH-wild GBMs, and NCOA4, OTUB1, SLC11A2, TF, TP53 and ZEB1 were downregulated in IDH-wild GBMs (Figure 3D). As the WHO grade increased, the expression of *BECN1, TP53, NFE2L2, SLC1A5, HSPB1*, and *FTL* increased, while the expression of *GOT1, NFS1, SLC11A2, SLC7A11*, and *TF* decreased.

### Prognostic value of ferroptosis regulators in glioma patients

**Figure 4:**
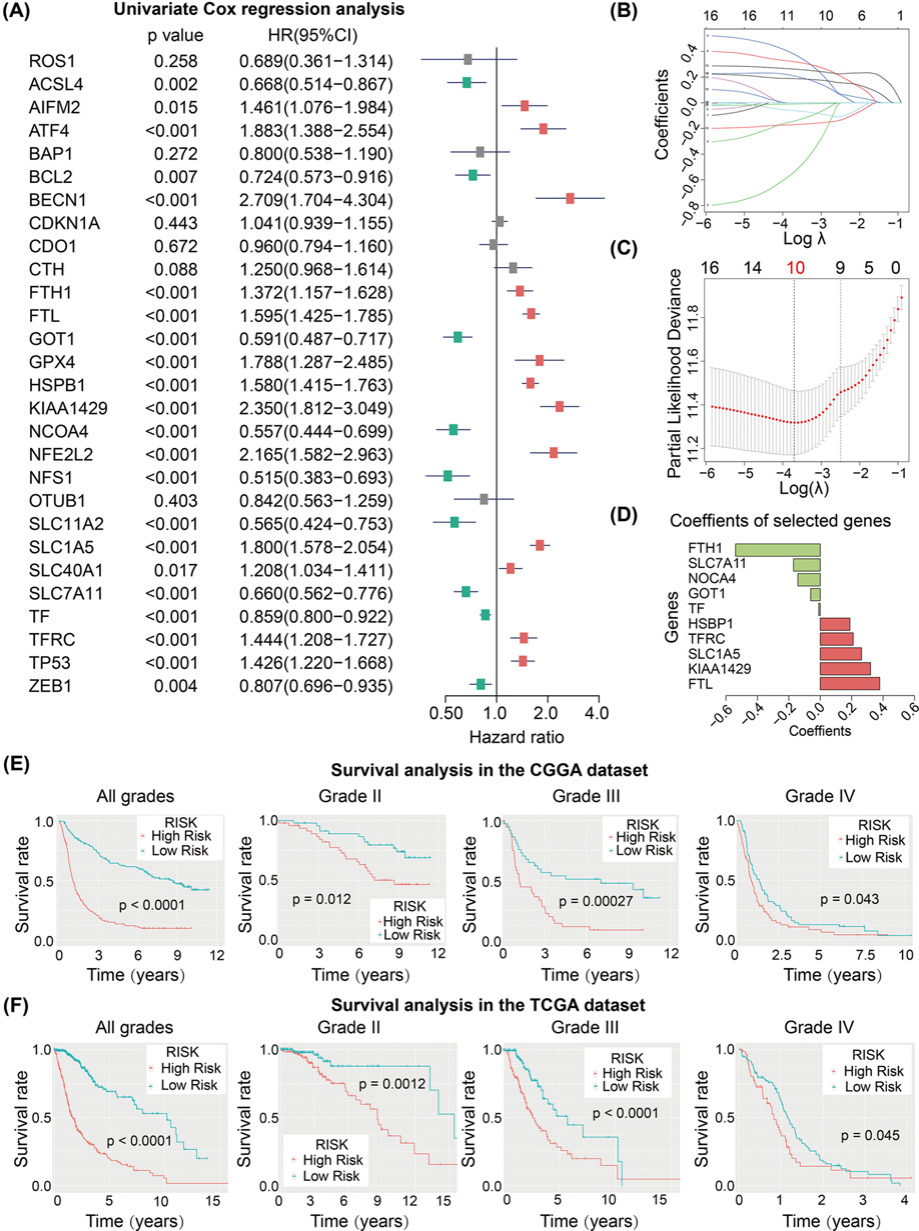
Establishment of the FRSig by multiple processes (**A**) Forest plot presents the results of univariate Cox regression of the 28 FRGs in the CGGA cohort. (**B–D**) Establishment of the FRSig using Lasso Cox regression method, and counting the minimum criteria (λ) (B,C) and corresponding coefficients (D). (**E**,**F**) Kaplan–Meier curves showed that the FRSig could identify higher risk gliomas in all grade gliomas and each grade gliomas in the CGGA (E) and TCGA (F) datasets.

Due to the significant associations between ferroptosis regulators and clinicopathological features of gliomas, we further investigated the prognostic values of these ferroptosis regulators using univariate Cox regression analysis in the CGGA dataset (Figure 4A). In total, 22 ferroptosis regulators were identified as associated with the overall survival (OS) of glioma patients in the CGGA cohort. The gene expression of *ACSL4*, *BCL2*, *GOT1*, *NCOA4*, *NFS1*, *SLC11A2*, *SLC7A11*, *TF*, and *ZEB1* was positively associated with the OS of glioma patients, while the expression of *AIFM2*, *ATF4*, *BECN1*, *FTH1*, *FTL*, *GPX4*, *HSPB1*, *KIAA1429*, *NFE2L2*, *SLC1A5*, *SLC40A1*, *TFRC*, and *TP53* were negatively correlated with OS.

### Development and validation of the ferroptosis-related risk signature

Based on the 22 prognostic ferroptosis regulators identified, we developed an FRSig by performing Lasso Cox regression algorithm using the CGGA dataset (Figure 4B–D). Ten of the 22 prognostic ferroptosis regulators were selected to develop the risk signature and the expression values. The regression coefficients of the ten FRGs were used to calculate a ferroptosis-related risk score for each patient in the CGGA dataset. Glioma patients with risk scores greater than the median value were defined as high-risk glioma patients, and they showed worse OS rate and time across all glioma grades and for each single glioma grade in the CGGA dataset (Figure 4E). To further validate the prognostic value of the FRSig, risk scores were also calculated for glioma patients in the TCGA dataset using the same formula. Similarly, the FRSig could distinguish glioma patients with different clinical outcomes for all grades of glioma or for each single grade (Figure 4F).

Furthermore, we explored the mRNA expression levels of *SLC1A5* and *HSPB1* in the GEPIA website and the protein expression levels using clinical samples between normal brain tissues and gliomas. The results showed that *SLC1A5* was up-regulated in gliomas compared with normal brain tissues in both mRNA and protein levels (Supplementary Figure S1A,B), and HSPB1 was also overexpressed in glioma samples (Supplementary Figure S1C,D).

### Functional enrichment and GSEA

**Figure 5:**
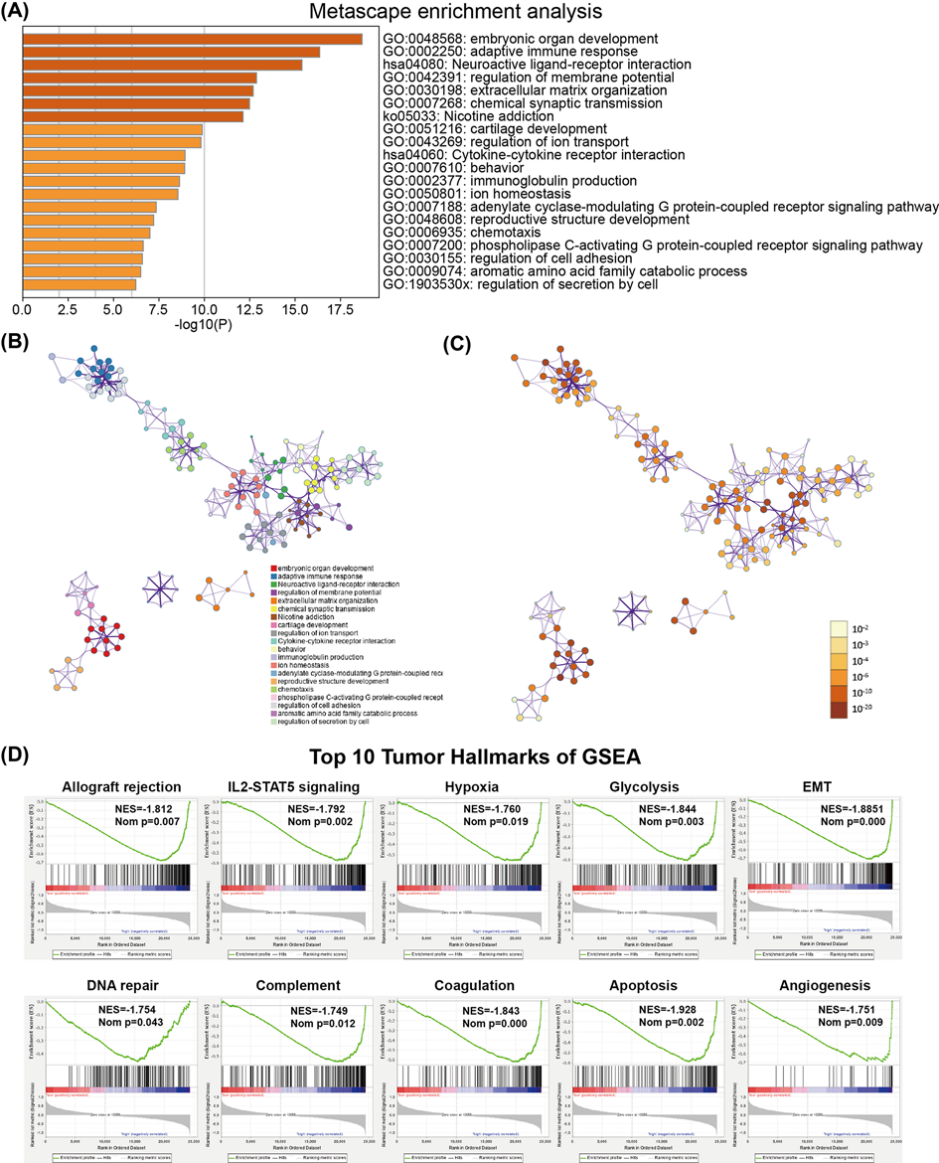
Functional enrichment analysis reveals the correlative biological process or pathway of FRSig (**A–C**) GO and KEGG pathway analysis results were visualized on the Metascape tool website. Bar plot (A) of enriched terms in the high-risk glioma subgroup (colored according to *P*-value), network of enriched terms colored according to (B) cluster ID and (C) *P*-value. (**D**) Top ten tumor hallmarks enriched in the high-risk glioma subgroup by GSEA.

To investigate the underlying biological functions which define the survival of glioma patients, we identified genes that were significantly differentially expressed between high- and low-risk glioma patients, and then annotated their function using GO pathway analysis for biological processes using the Metascape website. The results indicated that these DEGs were enriched in several immune-related biological processes, including the humoral immune response, production of molecular mediators of the immune response, and immunoregulatory interactions between lymphoid and non-lymphoid cells (Figure 5A–C). Furthermore, our FRSig was also associated with the development of the skeletal system, extracellular matrix organization, regulation of ion transport, and regulation of cell secretion (Figure 5A–C).

Furthermore, GSEA revealed that gene hallmarks, including allograft rejection (NES = 1.812, normalized *P*=0.007), IL2-STAT5 signaling (NES = 1.792, normalized *P*=0.002), hypoxia (NES = 1.76, normalized *P*=0.019), glycolysis (NES = 1.844, normalized *P*=0.003), DNA repair (NES = 1.754, normalized *P*=0.043), complement (NES = 1.749, normalized *P*=0.012), coagulation (NES = 1.843, normalized *P*<0.001), and apoptosis (NES = 1.928, normalized *P*=0.002), were significantly associated with high-risk glioma patients (Figure 5D).

### Clinical and molecular features and ferroptosis-related risk signature

**Figure 6:**
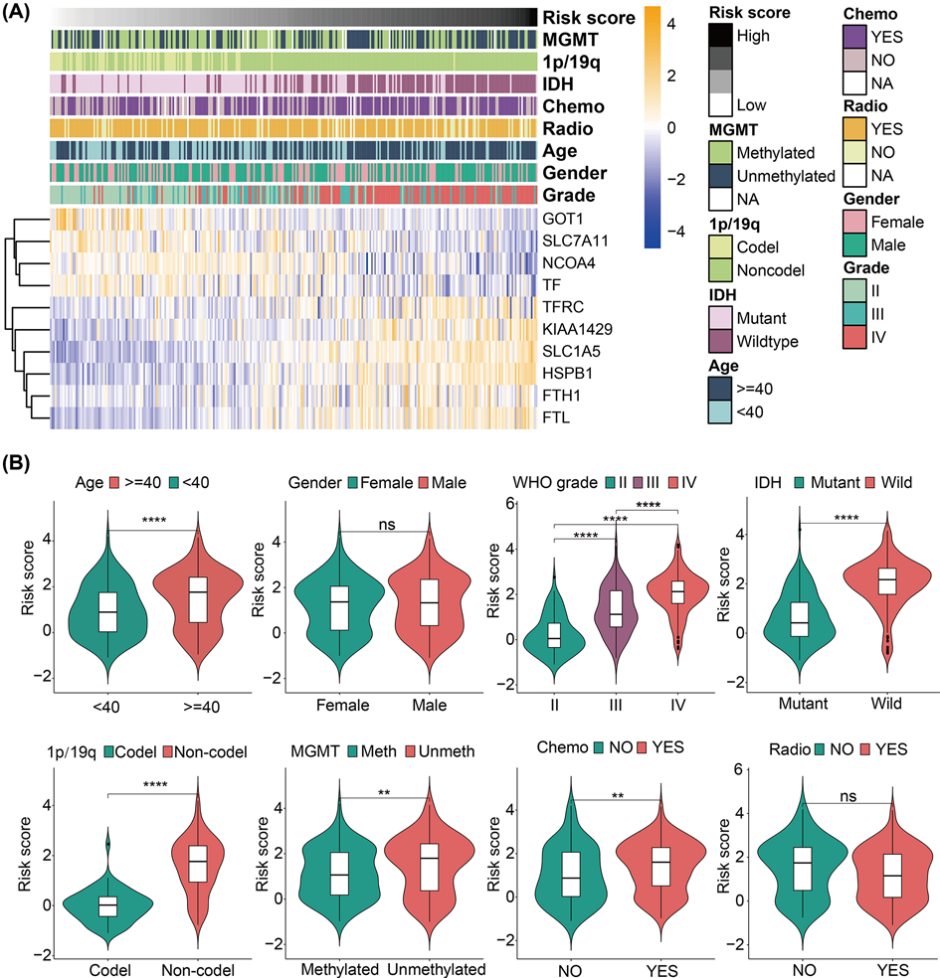
Clinicopathological relationships of FRSig in gliomas (**A**) Heatmap shows the correlations among the expressions of the ten screened-out FRGs and clinicopathological features of gliomas in the CGGA discovering dataset (ordered by the risk score form low to high). (**B**) The violin plots showed the statistical significance of the risk score between/among different clinicopathological features (including age, gender, WHO grade, IDH mutation status, 1p/19q co-deletion status, MGMT methylation status, chemotherapy, and radiotherapy status). ns *P*>0.05, *P<0.05, **P<0.01, ***P<0.001, ****P<0.001.

To evaluate the prognostic role of FRSig, we investigated the associations between the FRSig and the clinical and molecular features of glioma patients. We designed a heatmap to illustrate the relationship between FRG expression, clinical and molecular features, and the risk scores in the CGGA dataset (Figure 6A). Next, violin plots were used to compare the risk score levels between glioma patients from different clinical and molecular subgroups. As shown in Figure 6B, higher risk scores were significantly associated with older age, higher WHO grades, IDH wildtype mutation status, absence of 1p/19q co-deletion, MGMT unmethylated status, and chemotherapy-treated glioma patients, while risk scores were not associated with sex or radiotherapy status.

### Characterization of immune infiltration and ferroptosis-related risk signature

**Figure 7:**
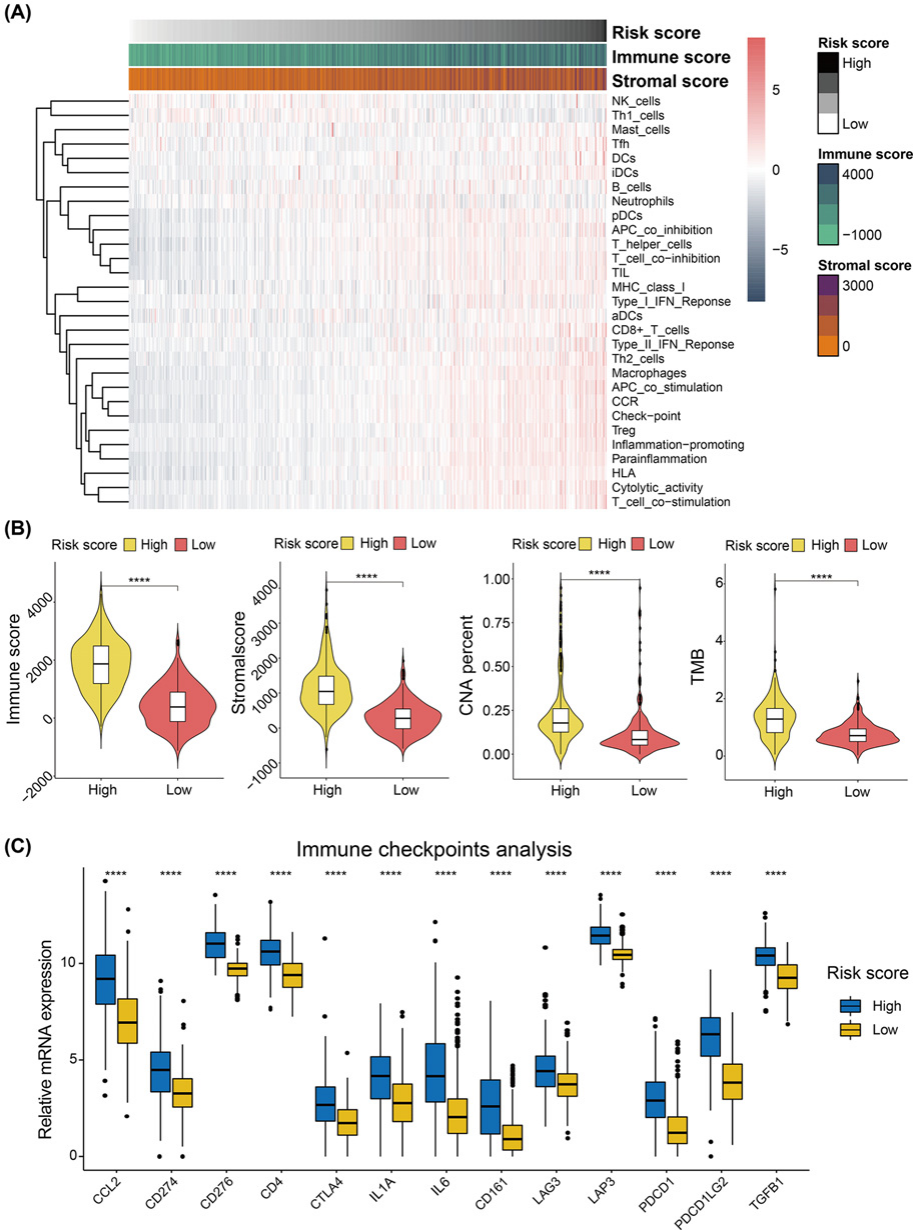
The immune-related analysis of FRSig in gliomas (**A**) Heatmaps represent the immune cell infiltrations and immune features associated with the risk score (ordered from low to high). (**B**) The immune score, stromal score, CNA burden, and TMB levels were significantly distinct statistically between low- and high-risk glioma subgroups. (**C**) The 13 well-known immune checkpoint genes were differentially expressed between low- and high-risk glioma subgroups. Abbreviation: CNA, copy number alteration. *P<0.05, **P<0.01, ***P<0.001, ****P<0.0001.

Based on the results of enrichment analysis, we found that the FRSig was associated with the immune response process, thus we speculated that our FRSig might be associated with the cancer immune features, which would help guide the application of immunotherapy to glioma in the future. Risk scores were significantly positively associated with the immune and stromal scores, and most relevant immune cell infiltrations or immune features calculated by ssGSEA algorithm were significantly correlated with the risk score (Figure 7A). Our results indicated that glioma patients with higher risk scores correlated with higher immune scores, stromal scores, CAN percent, and tumor mutation burden (TMB) (Figure 7B). Furthermore, immune checkpoint-related genes, including C–C motif chemokine ligand 2 (CCL2), CD274 (also known as PD-L1), CD276, CD4, cytotoxic T-lymphocyte associated protein 4 (CTLA4), interleukin 1 α (IL1A), interleukin 6 (IL6), killer cell lectin like receptor B1 (CD161, also known as KLRB1), lymphocyte activating 3 (LAG3), leucine aminopeptidase 3 (LAP3), programmed cell death 1 (PD-1, also known as PDCD1), and programmed cell death 1 ligand 2 (PDCDLG2), and transforming growth factor β 1 (TGFB1), were also included in our analysis. Glioma patients with higher risk scores had higher levels of immune checkpoint mRNA expression (Figure 7C).

### Prognostic value of the FRSig

**Figure 8:**
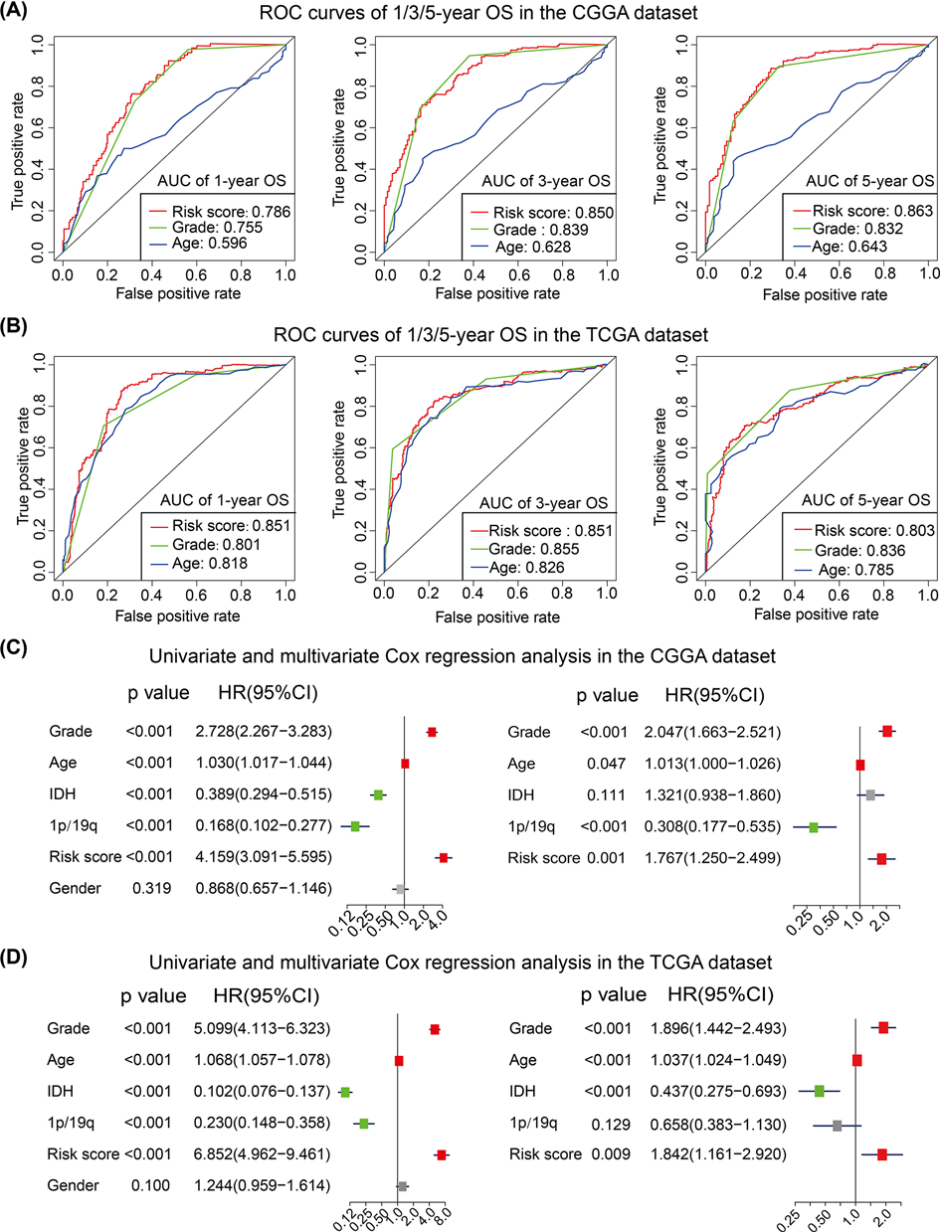
Identification of the independent prognostic role of FRSig in glioma (**A**,**B**) Time-dependent ROC curves of the risk score, age and WHO grade in predicting 1-, 3-, 5-year OS of glioma patients in the CGGA (A) and TCGA (B) datasets. (**C**,**D**) Univariate and multivariate Cox regression analyses of clinicopathological features in the CGGA (C) and TCGA (D) datasets.

To assess the prognostic and predictive values of the FRSig, ROC curves were used to evaluate the predictive accuracy of OS stratifying cases by age, WHO grade, and ferroptosis-related risk score. The risk score had a higher AUC value compared with stratification by age or WHO grade for the prediction of the 1-, 3-, and 5-year OS in the CGGA dataset (Figure 8A). The ROC curves performed in the TCGA dataset also revealed that the ferroptosis-related risk score could suitably predict the 1-, 3-, or 5-year OS of gliomas with all AUC values of more than 0.800 (Figure 8B).

Moreover, we performed univariate Cox regression to evaluate the prognostic roles of clinicopathological and molecular features (including age, sex, WHO grade, IDH mutant status, 1p/19q co-deletion status, and the ferroptosis-related risk score) in both the CGGA training and TCGA validation datasets. The molecular status of IDH mutation and 1p/19q co-deletion were protective factors, while older age, WHO grade, and the ferroptosis-related risk scores were risk factors for glioma patients in the CGGA dataset (Figure 8C). We then excluded the patient sex from the multivariate Cox regression analysis given it was not significantly associated with a prognostic role in the univariate Cox regression analysis. Multivariate Cox regression showed that age, WHO grade, 1p/19q co-deletion status, and the ferroptosis-related risk score were independent prognostic factors in the CGGA dataset (Figure 8C). Similar results were found in the TCGA validation dataset, whereby age, WHO grade and FRSig were identified as independent indicators of OS of glioma patients (Figure 8D).

### Establishment of a nomogram model based on the FRSig

**Figure 9:**
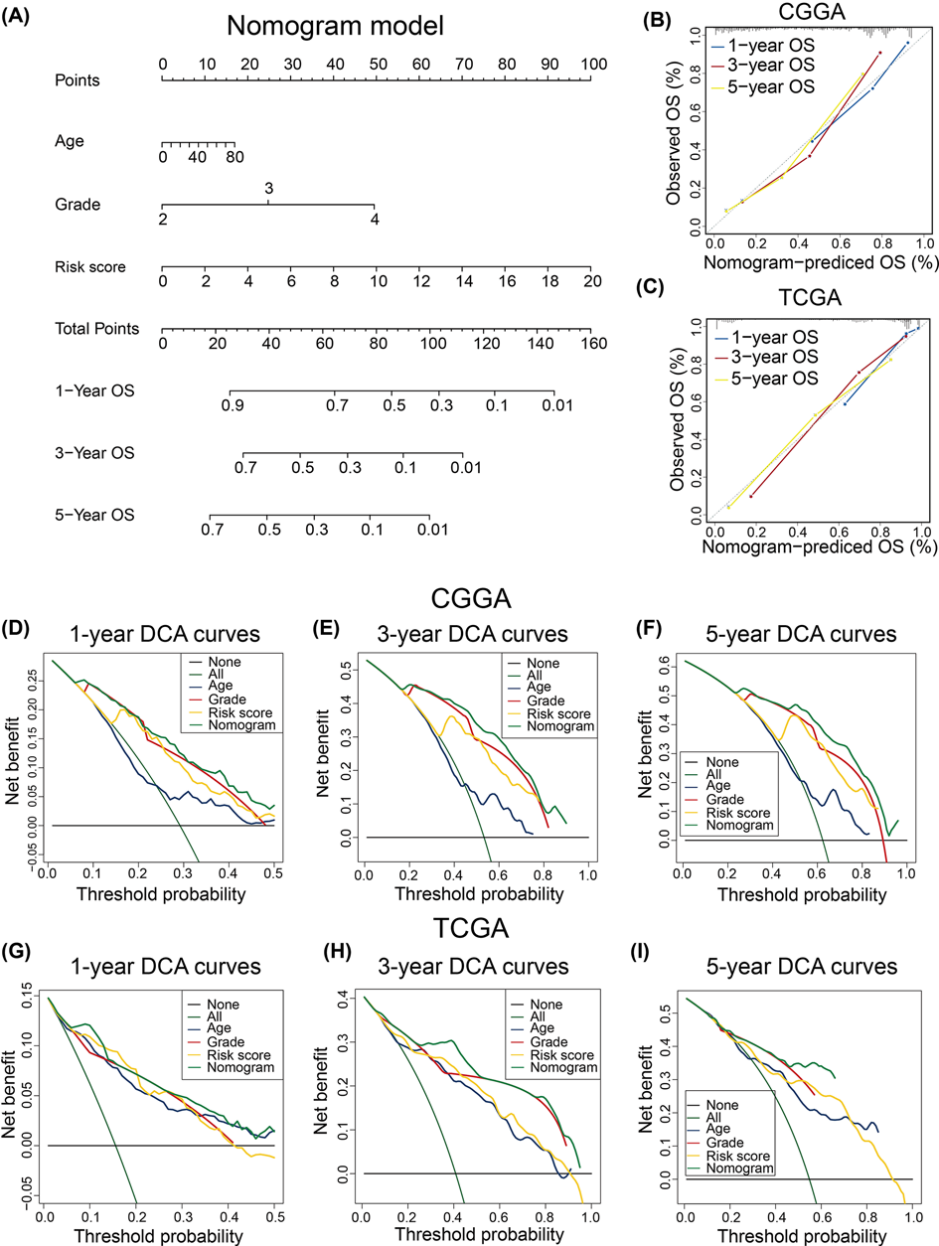
Establishment and validation of the FRSig-based nomogram model (**A**) The clinical nomogram model constructed in the CGGA glioma cohort. (**B**,**C**) The calibration curves in the CGGA discovering cohort (B) and TCGA validation cohort (C). (**D–F**) The DCA showed the net benefit of the nomogram model in predicting the 1-year (D), 3-year (E), and 5-year (F) OS of glioma patients in the CGGA discovery cohort. (**G–I**) The DCA showed the net benefit of the nomogram model in predicting the 1-year (G), 3-year (H), and 5-year (I) OS of glioma patients in the TCGA discovery cohort.

To further evaluate the clinical application of our FRSig, we constructed a nomogram prognostic model, based on the multivariate Cox regression results and the accessibility of clinicopathological factors in clinical diagnosis and treatment. We included the WHO grade, age, and FRSig as variables in the CGGA dataset (Figure 9A). In the CGGA cohort, calibration curves showed that the clinical nomogram model could precisely predict the 1-, 3-, and 5-year OS of patients with gliomas (C-index = 0.762) (Figure 9B), and the predictive accuracy of this nomogram was well validated in the TCGA cohort (C-index = 0.846) (Figure 9C).

Furthermore, we used DCA curves to quantify the relevant clinical benefit of the FRSig-based nomogram model. In comparison with the FRSig, age, and WHO grade, glioma patients obtained greater net benefit with a wider threshold probability for 1-, 3-, and 5-year OS prediction (Figure 9D–F) using the nomogram in the CGGA dataset. The associated DCA curves were also plotted for TCGA cohort (Figure 9G–I) and similar results were obtained, indicating that the nomogram could stably enhance the clinical benefits of glioma patients.

## Discussion

It has been reported that ferroptosis plays a crucial role in tumor initiation, progression, and intratumor cell infiltration, but these properties have not achieved a prominent role in clinical application. This emphasizes our work to construct the FRSig prognostic model and its importance in predicting clinical outcomes of glioma patients. In the present study, we assessed aberrant gene expression and its association with clinicopathological features in glioma patients and constructed the FRSig based on ten FRGs in TCGA cohort, which was validated in an independent CGGA cohort. This FRSig could stratify glioma patients into groups having distinct clinical outcomes and immune microenvironment infiltrations. Thus, the novel identified FRSig suitably reflected the status of the glioma microenvironment and acted as a strong prognostic and predictive biomarker.

Our results indicated that FRGs expression was strongly correlated with clinicopathological features and the FRSig significantly associated with the OS of glioma patients. Furthermore, the FRSig was constructed based on FRGs and reflected underlying correlations with microenvironment tumor cell infiltration, immune checkpoint gene expression, TMB, and copy number alteration (CNA) percentage. Given the predictive ability of TMB associated with the response to anti-PD-1/PD-L1 immunotherapy therapy, we reasonably speculated that glioma patients having higher FRSig scores should receive immune checkpoint inhibitor therapy. Considering the strong positive correlation identified found between the FRSig score and the expression of 13 identified immune checkpoint genes in glioma patients, these results might support the practical application of FRSig in precision immunotherapy of glioma patients in the future.

For the last few years, many researchers have focused their attention on the immune microenvironment of solid tumors to better understand the interaction between tumor cells and non-neoplastic cells, and ferroptosis has proven to be important in molding the tumor microenvironment. For example, tumor cells in the state of ferroptosis may shape cancer cell immunogenicity by releasing high mobility group box 1 (HMGB1) in an autophagy-dependent manner [19,20]. Furthermore, it has also been reported that immunotherapy-activated CD8^+^ T cells could induce cancer cell ferroptosis by decreasing SLC7A11 transcriptional expression and downstream secretion of IFN-γ, and consequently enhance the binding of signal transducer and activator of transcription 1 (STAT1) to the SLC7A11 transcription start site [21]. However, the elucidation of the comprehensive and intricate relationship between ferroptosis and antitumor immunity requires additional study to unravel and our study could offer clues for further experiments.

We constructed a stable and powerful prognostic model by using univariate Cox regression combined with a Lasso regression algorithm. Our approach may be considered as an excellent strategy for processing high-dimensional gene expression data, and the FRSig performed well in an independent external patient cohort. Nevertheless, there were several objective limitations to be considered in this study. The primary limitation was that given the retrospective nature of the glioma cohorts, our findings require validation in a prospective cohort. In addition, several new biomarker genes were identified in the FRSig, and to further support our results, their expression and biological functions need to be studied by *in vivo* or *in vitro* experiments.

In conclusion, we constructed a new ferroptosis-related risk model that not only presented accurate prognostic ability but also reflected the level of immune cell infiltration of the microenvironment in glioma patients. This model possesses potential value for clinical application. For example, glioma patients receiving surgical treatment could be prognostically classified by calculating the FRSig risk scores, and as the scores represent the status of glioma immune microenvironment, this may be used to guide the application of immunotherapy in the clinical setting.

## Supplementary Material

Supplementary Figure S1

## Data Availability

The original contributions presented in the study are included in the CGGA (http://www.cgga.org.cn/), the University of California, Santa Cruz Xena browser (UCSC Xena; https://xenabrowser.net/datapages/), and the GDC (https://portal.gdc.cancer.gov/).

## Abbreviations

AUC: area under the curve
CGGA: Chinese Glioma Genome Atlas
CNA: copy number alteration
DCA: decision curve analysis
DEG: differentially expressed gene
FRG: ferroptosis-related gene
FRSig: ferroptosis-related signature
GBM: glioblastoma
GDC: Genomic Data Commons Data Portal
GO: Gene Ontology
GSEA: gene set enrichment analysis
HR: hazard ratio
IDH: isocitrate dehydrogenase
Lasso: least absolute shrinkage and selection operator
OS: overall survival
ROC: receiver operating characteristic curve
ssGSEA: single-sample gene set enrichment analysis
TCGA: The Cancer Genome Atlas
TMB: tumor mutation burden
TPM: transcripts per kilobase million
WHO: World Health Organization
