# Supplementary figures and images for: Ferroptosis-related gene signature correlates with the tumor immune features and predicts the prognosis of glioma patients
Source: Biosci Rep. 2021 Dec 7;41(12):BSR20211640. doi: 10.1042/BSR20211640 (PMC8655507; doi:10.1042/BSR20211640)

A

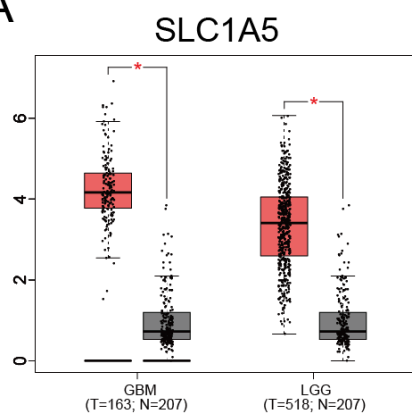

B

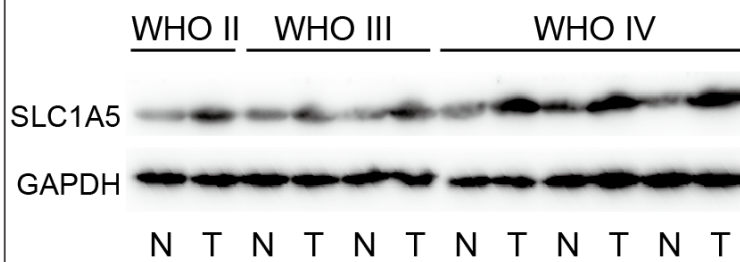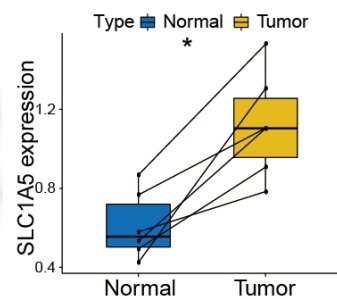

C

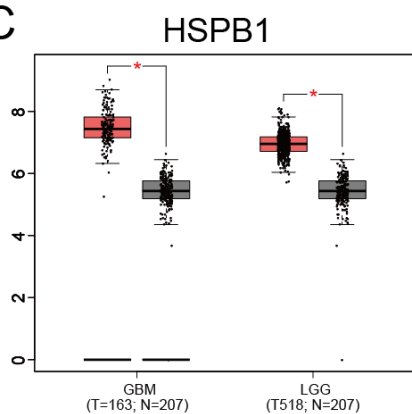

D

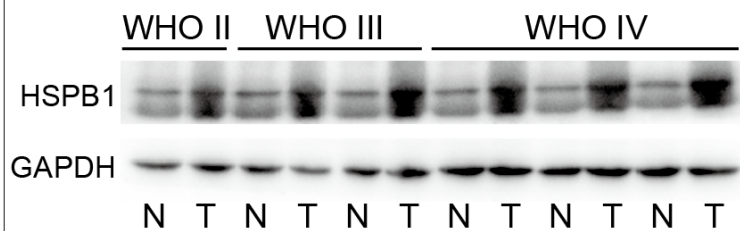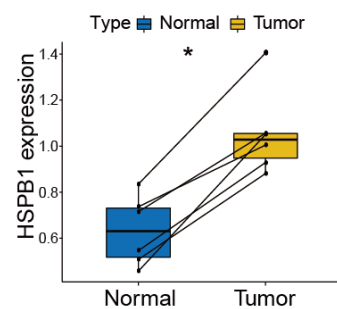

Supplement: Supplementary Figure S1 [file BSR-2021-1640_supp.pdf]
